# The complexity of visual dysfunction in patients with pseudoxanthoma elasticum

**DOI:** 10.1038/s41433-021-01858-7

**Published:** 2021-12-03

**Authors:** Peter Charbel Issa, Kristina Hess

**Affiliations:** 1 Oxford Eye Hospital, Oxford University Hospitals NHS Foundation Trust, Oxford, United Kingdom; 2 Nuffield Laboratory of Ophthalmology, Nuffield Department of Clinical Neurosciences, University of Oxford, Oxford, United Kingdom; 3 Division of Epidemiology and Clinical Applications, National Eye Institute, National Institutes of Health, Bethesda, MD USA

**Keywords:** Retinal diseases, Outcomes research

Pseudoxanthoma elasticum (PXE) is a multisystem disease characterized by calcification of elastic fibers. The eye is one of the most consistently and most severely affected organs, with vision loss being a major concern for PXE patients [1, 2]. Hence, a good understanding of the ocular structural changes and complex visual deficits in PXE is important for determining ocular disease severity, patient counseling and defining outcome measures and patient stratification in clinical trials.

## Structural alterations

The initial alteration in eyes of PXE patients is the multifocal, punctate calcification of Bruch’s membrane (BrM) at the posterior pole. The clinical finding at this stage is a peau d’orange appearance that becomes increasingly confluent at the posterior pole, from where it spreads centrifugally [1, 3, 4]. The BrM is a barrier between the choriocapillaris and the retinal pigment epithelium (RPE)-photoreceptor complex. BrM calcification is thought to increase resistance for the physiological metabolic exchange between the highly vascular choroid and the RPE, which eventually may result in secondary RPE dysfunction and degeneration. Due to its support-functions for the retina [5], such RPE changes may lead to impairment of the visual cycle, retinal adherence, and metabolic support for photoreceptors and the choriocapillaris. Subsequent chorioretinal atrophy may be seen as the natural endpoint of BrM calcification [6].

Furthermore, calcified BrM that lacks elasticity may develop cracks which are the structural correlate for angioid streaks [7]. Only very wide streaks with associated degeneration of the overlying RPE may cause focal functional loss, which usually remains unnoticed when located outside the fovea. Angioid streaks are a risk factor for secondary complications that are not directly related to the initial calcification process, such as choroidal neovascularization (CNV) or acute retinopathy [1, 8].

CNV membranes may either remain below the RPE (type 1) or can break through the RPE into the subretinal space (type 2). Exudation and/or subretinal hemorrhages may eventually lead to subretinal fibrosis with atrophy of the overlying photoreceptors. However, it is possible that not all CNVs are detrimental: non-exudative (quiescent) occult CNVs might play a role in ensuring metabolic support for the RPE and outer retina when this is not sufficiently provided anymore by the choroid due to an increased BrM barrier [6, 9], possibly slowing down development of atrophy [10, 11].

Acute retinopathy has been interpreted as an autoimmune process triggered by ocular antigens that are normally hidden from the immune system [8]. Its manifestation may be most pronounced along angioid streaks where such antigens are exposed. Acute retinopathy shares phenotypic similarities with multiple evanescent white dot syndrome, however, the full phenotypic spectrum and actual frequency require further exploration. It may be self-limiting with (partial) recovery but may also be associated with relatively fast progression of subretinal fibrosis and RPE/photoreceptor atrophy [8].

Independent from chorioretinal atrophy, inner retinal neurons may also degenerate in PXE [12]. This was mainly observed in eyes with optic nerve head drusen (ONHD) which commonly occur in eyes of PXE patients [1]. It is thought that ONHD may cause axonal damage due to mechanical stress in the space-limited environment of the optic disc. Thinning of the inner retina also seems to occur in eyes without ONHD, but further studies are required to explain this finding [12].

Cerebral vascular disease is common in PXE patients (17% in a recent study [13]) and may lead to impaired visual information processing, although the visual relevance of cerebral vascular events has not yet been investigated for PXE.

## Visual deficits in PXE patients

Visual acuity is a commonly used outcome measure in clinical trials. It is typically well preserved in the majority of PXE patients during the first four decades of life, after which vision increasingly deteriorates [14]. The structural alteration underlying permanent reduction in visual acuity is loss of foveal photoreceptors. This usually develops with atrophy of the RPE due to advanced BrM damage or subretinal fibrosis, or may occur secondary to acute retinopathy.

Dark adaptation may be impaired in PXE patients, independent from changes in visual acuity [15]. Explanations for impaired dark adaptation include an increased diffusion barrier for vitamin A caused by an altered BrM and impaired adherence of photoreceptors to the RPE, reduced turnover in the visual cycle due to functional impairment of RPE, or functional and/or degenerative changes of rod photoreceptors.

Contrast vision and low luminance visual acuity can also be impaired independent from dark adaptation ability; [15] the exact causes for these complex visual dysfunctions remain to be determined.

Finally, visual field loss may occur in topographic relation with photoreceptor dysfunction and loss, or with ganglion cell loss as a consequence of ONHD. These anatomic alterations as well as cerebral vascular disease may also lead to diffuse reduction of retinal sensitivity on visual field testing. Of note, peau d’orange as one of the hallmarks of the disease represents a rather early clinical finding (transition zone between non-calcified Bruch’s membrane and confluent calcification) [3] and is not expected to be associated with significant vision loss.

**Fig. 1:**
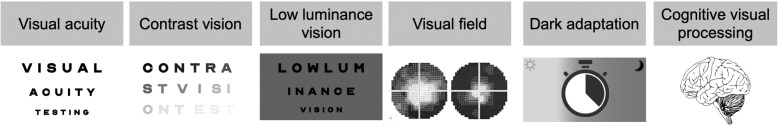
Visual functions that may be affected in patients with PXE.

The above-mentioned visual deficits (summarized in Fig. 1) may have variable effects on vision-related quality of life. The extent of each visual deficit varies between patients, resulting in a qualitative and quantitative heterogeneity of visual dysfunction. Direct conclusions on the vision-related quality of life based on structural and functional measures may be limited because perceived disease burden may depend on individual coping strategies, age, personal circumstances, and occupation. In addition, vision-related quality of life may also be affected by visual perceptions such as metamorphopsia, photopsia (e.g. shimmering lights associated with acute retinopathy) or Charles-Bonnet-Syndrome. These phenomena characteristically have a poor structure-function correlation and are challenging to quantify.

Importantly, visual acuity alone, which is most commonly used to assess visual function in practice as well as in clinical trials, is unlikely to reliably reflect the true vision-related disease burden. For instance, PXE patients with normal visual acuity may be unable to read the menu in a restaurant and unable to drive at night due to poor ambient illumination or low contrast lighting conditions. Vision standards for driving also include an adequately preserved visual field which may be affected by ONHD, even when visual acuity and photoreceptor function would be sufficient.

To summarize, PXE patients may present with a wide range of visual deficits that likely remain undetected on routine clinical testing. A combination of functional tests is necessary to reflect the heterogenic manifestation and severity of the disease. For clinical trials targeting BrM mineralization, strict inclusion/exclusion-criteria are warranted to minimize variability due to secondary alterations that are not directly related to BrM calcification.

## References

[1] Gliem M, De Zaeytijd J, Finger RP, Holz FG, Leroy BP, Charbel Issa P (2013). An update on the ocular phenotype in patients with pseudoxanthoma elasticum. Front Genet.

[2] Finger RP, Fenwick E, Marella M, Charbel Issa P, Scholl HP, Holz FG (2011). The relative impact of vision impairment and cardiovascular disease on quality of life: the example of pseudoxanthoma elasticum. Health Qual Life Outcomes.

[3] Charbel Issa P, Finger RP, Götting C, Hendig D, Holz FG, Scholl HP (2010). Centrifugal fundus abnormalities in pseudoxanthoma elasticum. Ophthalmology.

[4] Risseeuw S, van Leeuwen R, Imhof SM, Spiering W, Ossewaarde-van Norel J (2021). The natural history of Bruch’s membrane calcification in pseudoxanthoma elasticum. Ophthalmol Sci.

[5] Strauss O (2005). The retinal pigment epithelium in visual function. Physiol Rev.

[6] Gliem M, Müller PL, Birtel J, Hendig D, Holz FG, Charbel (2016). Frequency, phenotypic characteristics and progression of atrophy associated with a diseased Bruch’s membrane in pseudoxanthoma elasticum. Invest Ophthalmol Vis Sci.

[7] Charbel Issa P, Finger RP, Holz FG, Scholl HP (2009). Multimodal imaging including spectral domain OCT and confocal near infrared reflectance for characterization of outer retinal pathology in pseudoxanthoma elasticum. Invest Ophthalmol Vis Sci.

[8] Gliem M, Birtel J, Müller PL, Hendig D, Faust I, Herrmann P (2019). Acute retinopathy in pseudoxanthoma elasticum. JAMA Ophthalmol.

[9] Marques JP, Bernardes J, Geada S, Soares M, Teixeira D, Farinha C (2021). Non-exudative macular neovascularization in pseudoxanthoma elasticum. Graefes Arch Clin Exp Ophthalmol.

[10] Hess K, Gliem M, Charbel Issa P, Birtel J, Müller PL, von der Emde L (2020). Mesopic and scotopic light sensitivity and its microstructural correlates in pseudoxanthoma elasticum. JAMA Ophthalmol.

[11] Pfau M, Möller PT, Künzel SH, von der Emde L, Lindner M, Thiele S (2020). Type 1 choroidal neovascularization is associated with reduced localized progression of atrophy in age-related macular degeneration. Ophthalmol Retin.

[12] Hess K, Raming K, Charbel Issa P, Herrmann P, Holz FG, Pfau M. Inner retinal degeneration associated with optic nerve head drusen in pseudoxanthoma elasticum. Br J Ophthalmol. 2021. 10.1136/bjophthalmol-2021-320088. 10.1136/bjophthalmol-2021-320088 34670750

[13] Kauw F, Kranenburg G, Kappelle LJ, Hendrikse J, Koek HL, Visseren FLJ (2017). Cerebral disease in a nationwide Dutch pseudoxanthoma elasticum cohort with a systematic review of the literature. J Neurol Sci.

[14] Risseeuw S, Ossewaarde-van Norel J, Klaver CCW, Colijn JM, Imhof SM, van Leeuwen R (2019). Visual acuity in pseudoxanthoma elasticum. Retina.

[15] Hess K, Gliem M, Birtel J, Müller P, Hendig D, Andrews C (2020). Impaired dark adaptation associated with a diseased bruch membrane in pseudoxanthoma elasticum. Retina.

